# Motility-related protein (MRP-1/CD9) and KAI1/CD82 expression inversely correlate with lymph node metastasis in oesophageal squamous cell carcinoma

**DOI:** 10.1038/sj.bjc.6690186

**Published:** 1999-03

**Authors:** S Uchida, Y Shimada, G Watanabe, Z Gang Li, T Hong, M Miyake, M Imamura

**Affiliations:** Department of Surgery and Surgical Basic Science, Graduate School of Medicine, Kyoto University, 54-Syogoin kawara-cho, Sakyoku, Kyoto 606-8507, Japan; Department of Thoracic Surgery, Kitano Hospital, Tazuke Kofukai Medical Research Institute, Osaka, 530, Japan

**Keywords:** oesophageal carcinoma, MRP-1/CD9, KAI1/CD82

## Abstract

Although the mechanisms of action of the transmembrane superfamilies, motility-related protein-1 (MRP-1/CD9) and KAI1/CD82, are not well known, they are reported to suppress the metastasis of several kinds of cancers. The suppression of cell motility by MRP-1/CD9 may cause suppression of the metastasis. As we could not find any reports concerning the expression of MRP-1/CD9 and KAI1/CD82 in oesophageal cancers we investigated their expression in oesophageal specimens. We conducted immunohistochemical staining for MRP-1/CD9 against 108 cases of oesophageal squamous cell carcinoma using anti-MRP-1/CD9 monoclonal antibody M31-15, and for KAI1/CD82 against 104 cases using anti-KAI1/CD82 monoclonal antibody C33. To investigate the gradual expression of MRP-1/CD9 and KAI1/CD82, 24 oesophageal dysplasias were immunohistochemically stained using the same method and then investigated.

The expression of both MRP-1/CD9 and KAI1/CD82 were positive on the cell membranes of normal oesophageal epithelial cells, but reduced or negative in the cancer cells. Reduced MRP-1/CD9 expressions significantly correlated to tumour depth (*P* = 0.0009). We found a significantly greater number of reduced or negative expression of MRP-1/CD9 and KAI1/CD82 in lymph node metastatic cases (*P* = 0.0003 and *P* = 0.0129, respectively), but not in distant metastatic cases. The 5-year survival rate of MRP-1/CD9-negative and reduced patients was significantly worse than those of positive patients (*n* = 108, curative cases, RO). Few cases remained KAI1/CD82-positive (9.6%; 10/104) in oesophageal cancer. Twenty (83.3%) and twenty-two (91.7%) cases out of 24 dysplasias were defined as KAI1/CD82-positive and MRP-1/CD9-positive, respectively. The decrease in MRP-1/CD9 and KAI1/CD82 expression may facilitate lymph node metastasis in oesophageal squamous cell carcinomas. Knowing the status of the expression of MRP-1/CD9 appears helpful in predicting the prognosis for each patient. © 1999 Cancer Research Campaign


*British Journal of Cancer* (1999) **79**(7/8), 1168–1173
© 1999 Cancer Research Campaign
Article no. bjoc.1998.0186

### Motility-related protein (MRP-1/CD9) and KAI1/CD82 expression inversely correlate with lymph node metastasis in oesophageal squamous cell carcinoma

**S Uchida[1], Y Shimada[1], G Watanabe[1], Z Gang Li[1], T Hong[1], M Miyake[2] and M Imamura[1]**

[1]Department of Surgery and Surgical Basic Science, Graduate School of Medicine, Kyoto University, 54-Syogoin kawara-cho, Sakyoku, Kyoto 606-8507, Japan; [2]Department of Thoracic Surgery, Kitano Hospital, Tazuke Kofukai Medical Research Institute, Osaka 530, Japan

**Summary** Although the mechanisms of action of the transmembrane superfamilies, motility-related protein-1 (MRP-1/CD9) and KAI1/CD82, are not well known, they are reported to suppress the metastasis of several kinds of cancers. The suppression of cell motility by MRP-1/CD9 may cause suppression of the metastasis. As we could not find any reports concerning the expression of MRP-1/CD9 and KAI1/CD82 in oesophageal cancers we investigated their expression in oesophageal specimens. We conducted immunohistochemical staining for MRP-1/CD9 against 108 cases of oesophageal squamous cell carcinoma using anti-MRP-1/CD9 monoclonal antibody M31-15, and for KAI1/CD82 against 104 cases using anti-KAI1/CD82 monoclonal antibody C33. To investigate the gradual expression of MRP-1/CD9 and KAI1/CD82, 24 oesophageal dysplasias were immunohistochemically stained using the same method and then investigated.

The expression of both MRP-1/CD9 and KAI1/CD82 were positive on the cell membranes of normal oesophageal epithelial cells, but reduced or negative in the cancer cells. Reduced MRP-1/CD9 expressions significantly correlated to tumour depth ($P$ = 0.0009). We found a significantly greater number of reduced or negative expression of MRP-1/CD9 and KAI1/CD82 in lymph node metastatic cases ($P$ = 0.0003 and $P$ = 0.0129, respectively), but not in distant metastatic cases. The 5-year survival rate of MRP-1/CD9-negative and reduced patients was significantly worse than those of positive patients ($n$ = 108, curative cases, RO). Few cases remained KAI1/CD82-positive (9.6%; 10/104) in oesophageal cancer. Twenty (83.3%) and twenty-two (91.7%) cases out of 24 dysplasias were defined as KAI1/CD82-positive and MRP-1/CD9-positive, respectively. The decrease in MRP-1/CD9 and KAI1/CD82 expression may facilitate lymph node metastasis in oesophageal squamous cell carcinomas. Knowing the status of the expression of MRP-1/CD9 appears helpful in predicting the prognosis for each patient.

**Keywords:** oesophageal carcinoma; MRP-1/CD9; KAI1/CD82

Metastasis of cancer cells is believed to be influenced by many factors which progress through several stages as cancer cells move from a primary lesion to metastatic sites. One of those factors, cell motility, plays an important role in the progression, especially after the extravasation. Motility-related protein 1 (MRP-1/CD9), which belongs to the transmembrane 4 superfamily of membrane proteins, is considered to inhibit cell motility (Miyake et al, 1991). It has been demonstrated that the cells which expressed MRP-1/CD9 by transfection showed suppressed cell motility in vitro (Ikeyama et al, 1993). An inverse correlation was found between MRP-1/CD9 expression and metastases in breast cancer (Miyake et al, 1995), and reduced *MRP-1/CD9* gene expressions resulted in poor prognoses in non-small cell lung cancer (Higashiyama et al, 1995). KAI1/CD82 is also one of the transmembrane 4 superfamily of membrane proteins which has similar effects, mediating metastasis and correlating positively with good prognosis in patients with non-small cell lung cancer (Adachi et al, 1996) and inversely with metastasis in prostatic cancers (Dong et al, 1995; Dong, 1996). There were no studies of MRP-1/CD9 and KAI1/CD82 in oesophageal cancer. In this paper, we investigated expressions of MRP-1/CD9 and KAI1/CD82 in oesophageal cancer.

*Received 23 October 1997*
*Revised 9 March 1998*
*Accepted 30 June 1998*

*Correspondence to:* S Uchida

### MATERIALS AND METHODS

### Clinical materials

Tissues, whose expressions of MRP-1/CD9 and KAI1/CD82 were examined, were obtained from oesophageal cancer specimens of 108 and 104 patients, respectively, who underwent oesophagectomy at our institution, from June 1987 to December 1995. The operative techniques were as previously described (Imamura et al, 1987). Some samples were not available to conduct immunohistochemical staining, therefore the number of the cases examined in MRP-1/CD9 and KAI1/CD82 was not identical. All resected tumours were microscopically examined to identify histologic type, extent and mode of cancer invasion, and metastasis to lymph nodes. In some cases lymphatic invasion could be seen in the apparent absence of lymph node metastases. Considering this, we investigated the correlation between the expression of MRP-1/CD9 and lymphatic invasion in 98 out of 108 patients. Nine patients were excluded because lymphatic invasion could not be conclusively determined.

Histologically, all of the patients had squamous cell carcinoma. No adenosquamous carcinoma or small cell variants were found. Tumour staging was based on the pTNM pathological classification system (Hermanek et al, 1987). The 5-year survival rates of all patients who received curative resections were examined.

Specimens were fixed in a 10% formaldehyde solution and embedded in paraffin. Four-micrometre sections were cut and mounted on glass slides.

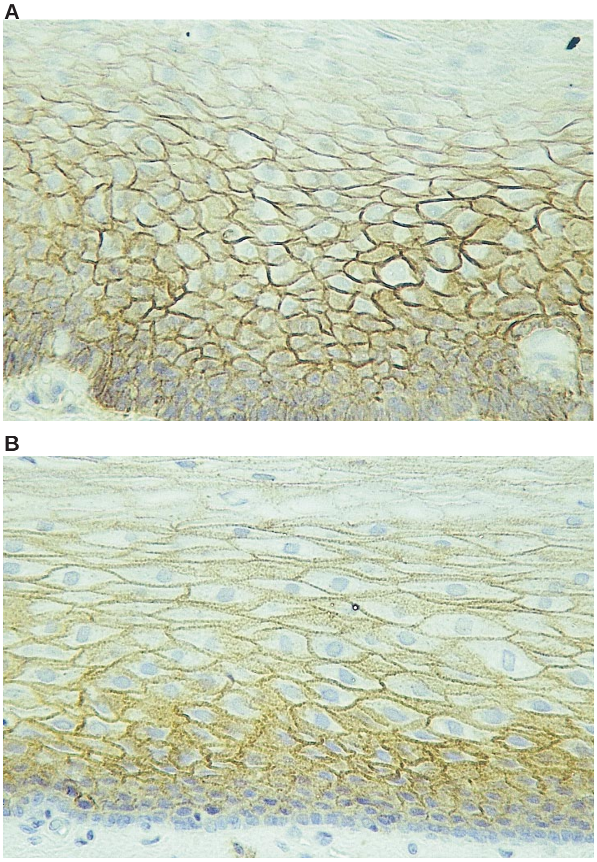

**Figure 1** (**A, B**) The immunohistochemical stainings of MRP-1/CD9 and KAI1/CD82 on oesophageal epithelium using antibody M31-15 and C33, respectively. Both molecules were detected positively by staining on normal oesophageal epithelial cellular membranes (magnification: 1A, × 200; 1B, × 200)

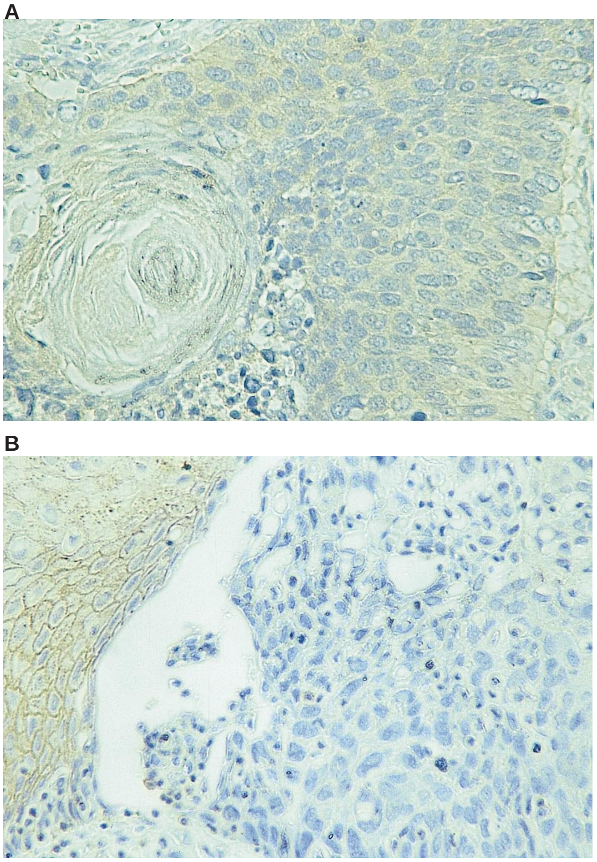

**Figure 2** (**A, B**) The immunohistochemical stainings of MRP-1/CD9 and KAI1/CD82 with the same methods on oesophageal squamous cell carcinoma. Neither molecule was detected on either cellular membrane or cytoplasma on carcinoma cells. Normal epithelial cellular membranes were stained as positive in Figure 2B. These stainings were defined as negative (magnification: 2A, × 200; 2B, × 200)

### Immunohistochemical staining and evaluation

Immunohistochemical staining was performed using the avidin–biotin method. Tissue sections were deparaffinized and rehydrated in water. Endogenous peroxidase was blocked with 0.3% hydrogen peroxide for 30 min. Sections were rehydrated and washed with phosphate-buffered saline (PBS). Slides were then placed in plastic Coplin jars containing 10% sodium citrate. Jars were heated in the microwave oven five times for 3 min each time. After heating, the jars were removed from the oven and allowed to cool for 20 min. After the slides were rinsed in PBS three times for 5 min, they were incubated with 1.5% normal horse serum in PBS for 30 min at room temperature to block non-specific antibody reaction. Sections were incubated overnight at 4°C with anti-MRP-1/CD9 monoclonal antibody M31-15 (Miyake et al, 1995) at a dilution of 1:20 in PBS containing 1% bovine serum albumin. After six rinses in PBS, sections were incubated for 40 min at room temperature with biotinylated anti-mouse immunoglobulin G, followed by six washes with PBS, and then reacted with an avidin–biotin system, using 0.03% 3,3′-diaminobenzide tetrahydrochloride for about 4 min as chromogen. Sections were counterstained with Mayer's haematoxylin. Negative controls were prepared by substituting normal mouse IgG for the primary antibody, and resulted in no detectable staining. In the case of KAI1/CD82 the immunostaining was conducted using the same methodology as stated above, with the exception that there was no antigen retrieval method by microwave oven. The antibody M31–15 was substituted with C33, a monoclonal antibody for KAI1/CD82 (Ueda et al, 1996) at a dilution of 1:100 in PBS containing 1% bovine serum albumin.

When more than 50% of the carcinoma cells in a given specimen were positively stained, the sample was classified as MRP-1/CD9-positive (+); when 5–50% were stained, as MRP-1/CD9-reduced (±); and when less than 5% were stained, as negative (–) (Miyake et al, 1995). When more than 10% of the carcinoma cells in a given specimen were positively stained, the sample was classified as KAI1/CD82-positive (+); and when less than 10% were stained, as KAI1/CD82-negative (–).

### Statistical analysis

Statistical analysis was performed with $\chi^2$ analysis and Fisher's exact test. Survival curves of the patients were calculated by Kaplan–Meier method and analysis was done by log-rank test.

### RESULTS

The expression of both MRP-1/CD9 and KAI1/CD82 appeared as positive on the cell membranes of the normal oesophageal epithelial cells (Figure 1A, MRP-1/CD9; Figure 1B, KAI1/CD82), and

*© Cancer Research Campaign 1999* 

**Table 1** Characteristics of 108 patients and correlation between the expression of MRP-1/CD9 and clinical classification

| | Result of immunostaining No. of patients (%) | | | | |
|---|---|---|---|---|---|
| | **MRP-1 (–)** | **MRP-1 (±)** | **MRP-1 (+)** | **Total** | ***P*-value** |
| Age (years) | 43 ~ 84 | | | | |
| (Mean age) | 64 | | | | |
| Sex (no. of patients) | | | | | |
| Male | 54 | 20 | 15 | 89 | |
| Female | 12 | 3 | 4 | 19 | $P = 0.7784$ |
| TNM clinical classification | | | | | |
| T – Primary tumour | | | | | |
| Tis | 0 (0) | 1 (33.3) | 2 (66.7) | 3 | |
| T1 | 10 (37.0) | 6 (22.2) | 11 (40.7) | 27 | |
| T2 | 17 (65.4) | 4 (15.4) | 5 (19.2) | 26 | |
| T3 | 26 (74.3) | 8 (22.9) | 1 (2.9) | 35 | |
| T4 | 13 (76.5) | 4 (23.5) | 0 (0) | 17 | $P = 0.0009$ |
| N – Regional lymph nodes | | | | | |
| N0 | 20 (48.8) | 6 (14.6) | 15 (36.6) | 41 | |
| N1 | 46 (68.7) | 17 (25.4) | 4 (6.0) | 67 | $P = 0.0003$ |
| M – Distant metastasis | | | | | |
| M0 | 53 (61.6) | 17 (19.8) | 16 (18.6) | 86 | |
| M1 | 13 (59.1) | 6 (27.3) | 3 (13.6) | 22 | $P = 0.6950$ |
| Stage | | | | | |
| Stage 0 | 0 (0) | 1 (33.3) | 2 (66.7) | 3 | |
| Stage I | 8 (44.4) | 1 (5.6) | 9 (50.0) | 18 | |
| Stage IIA | 7 (50.0) | 4 (28.6) | 3 (21.4) | 14 | |
| Stage IIB | 14 (77.8) | 3 (16.7) | 1 (5.6) | 18 | |
| Stage III | 24 (72.7) | 8 (24.2) | 1 (3.0) | 33 | |
| Stage IV | 13 (59.1) | 6 (27.3) | 3 (13.6) | 22 | $P = 0.0015$ |

Classification method is from *TNM Clinical Classification of the Oesophagus* 4th edn (1987).

**Table 2** Correlation between the expression of MRP-1/CD9 and lymph node metastasis and lymphatic invasion

| | Result of immunostaining No. of patients (%) | | | | |
|---|---|---|---|---|---|
| | **MRP-1 (–)** | **MRP-1 (±)** | **MRP-1 (+)** | **Total** | ***P*-value** |
| N – Regional lymph nodes | | | | | |
| N0 | 19 (51.4) | 6 (16.2) | 12 (32.4) | 37 | |
| N1 | 43 (70.5) | 14 (23.0) | 4 (6.6) | 61 | $P = 0.0035$ |
| Lymphatic invasion | | | | | |
| ly0 | 17 (53.1) | 5 (15.6) | 10 (31.3) | 32 | |
| ly1 | 45 (68.2) | 15 (22.7) | 6 (9.1) | 66 | $P = 0.0204$ |

N0, no regional lymph node metastasis; N1, regional lymph node metastasis; ly 0, no lymphatic invasions; ly 1, lymphatic invasions.

**Table 3** Correlation between lymph node metastasis and lymphatic invasion in the same 98 patients

| | N-Regional lymph nodes No. of patients | | | |
|---|---|---|---|---|
| | **N0** | **N1** | **Total** | ***P*-value** |
| lymphatic invasion | | | | |
| ly0 | 20 | 12 | 32 | |
| ly1 | 17 | 49 | 66 | $P = 0.004$ |

ly0, no lymphatic invasions; ly 1, lymphatic invasions.

 *© Cancer Research Campaign 1999*

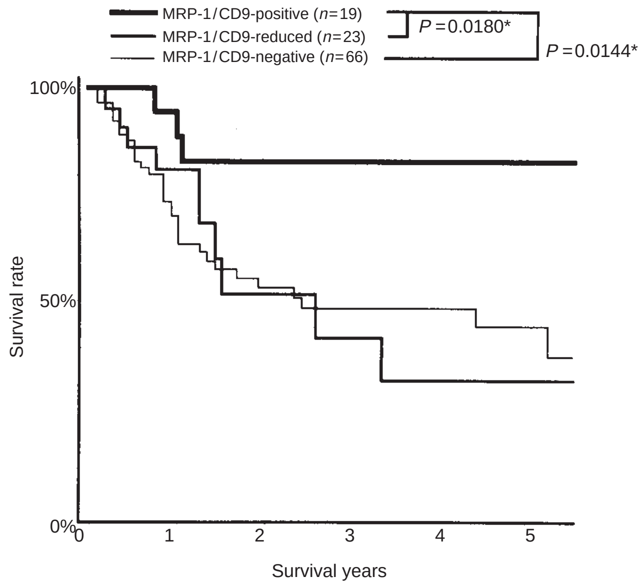

**Figure 3** The relation between the influence of MRP-1/CD9 and survival rates. Survival curves of the patients were calculated by Kaplan–Meier method. The 5-year survival rates of MRP-1/CD9-negative and MRP-1/CD9-reduced patients showed significantly worse prognoses than that of positive patients ($n$ = 108, curative cases). [a]log-rank test reduced on the membrane of the cancer cells (Figure 2A, MRP-1/CD9; Figure 2B, KAI1/CD82).

The number of reduced MRP-1/CD9 expressions significantly increased as tumours grew deeper ($P = 0.0009$). We found a significant inverse correlation between MRP-1/CD9 expression and lymph node metastasis ($P = 0.0003$), but not between MRP-1/CD9 expression and distant metastasis. Reduced expression of MRP-1/CD9 increased as the stage advanced (Table 1). In the 98 patients investigated, we found significant inverse correlation between MRP-1/CD9 expression and lymphatic invasion ($P = 0.0204$). In these same 98 patients the inverse correlation between MRP-1/CD9 expression and lymph node metastases was also confirmed ($P = 0.0035$) (Table 2). Correspondingly, the lymph node metastases correlated to lymphatic invasion ($P = 0.004$) (Table 3).

The survival rates of 108 patients who received curative resection (R0) were examined. (Seventeen stage IV patients did not have organ metastasis except for cervical lymph nodal metastases, which were resected.) The 5-year survival rates of MRP-1/CD9-negative and MRP-1/CD9-reduced patients were significantly worse than those of MRP-1/CD9-positive patients (Figure 3).

The expression of KAI1/CD82 was also inversely correlated to lymph node metastasis, but not to distant metastasis (Table 4). Few cases stained KAI1/CD82-positive (9.6%; 10/104) in oesophageal cancer, therefore, we examined oesophageal dysplasias to investigate whether KAI1/CD82 expression was preserved in them. We

**Table 4** Characteristics of 104 patients and correlation between the expression of KAI1/CD82 and clinical classification

| | | | | |
|---|---|---|---|---|
| Age (years) | 43 ~ 84 | | | |
| (Mean age) | 64 | | | |
| | **Result of immunostaining No. of patients (%)** | | | |
| | **KAI1 (–)** | **KAI1 (+)** | **Total** | ***P*-value** |
| Sex (no. of patients) | | | | |
| Male | 75 | 10 | 85 | |
| Female | 19 | 0 | 19 | $P = 0.2018$* |
| TNM clinical classification | | | | |
| T – Primary tumour | | | | |
| Tis | 3 (42.9) | 4 (57.1) | 7 | |
| T1 | 23 (88.5) | 3 (11.5) | 26 | |
| T2 | 24 (96.0) | 1 (4.0) | 25 | |
| T3 | 29 (96.7) | 1 (3.3) | 30 | |
| T4 | 15 (93.8) | 1 (6.2) | 16 | $P = 0.0003$ |
| N – Regional lymph nodes | | | | |
| N0 | 33 (80.5) | 8 (19.5) | 41 | |
| N1 | 61 (96.8) | 2 (3.2) | 63 | $P = 0.0129$* |
| M – Distant metastasis | | | | |
| M0 | 77 (90.6) | 8 (9.4) | 85 | |
| M1 | 19 (100) | 0 (0) | 19 | $P > 0.9999$* |
| Stage | | | | |
| Stage 0 | 3 (42.9) | 4 (57.1) | 7 | |
| Stage I | 14 (82.4) | 3 (17.6) | 17 | |
| Stage IIA | 11 (91.7) | 1 (8.3) | 12 | |
| Stage IIB | 18 (100) | 0 (0) | 18 | |
| Stage III | 31 (100) | 0 (0) | 33 | |
| Stage IV | 17 (89.5) | 2 (10.5) | 19 | $P = 0.0002$ |

Classification method is from *TNM Clinical Classification of the Oesophagus*, 4th Edn (1987). *Fisher's exact test.

*© Cancer Research Campaign 1999*

examined 24 dysplastic lesions which were taken from biopsied preoperative samples. Out of 24 dysplasias examined using the same immunohistochemical method, 20 (83.3%) cases of the dysplasia were defined as KAI1/CD82-positive. We then conducted immunohistochemical staining of MRP-1/CD9 on the same dysplasias, and 22 (91.7%) were defined as MRP-1/CD9-positive.

### DISCUSSION

We have shown that reduced expression of both transmenbrane proteins, MRP-1/CD9 and KAI1/CD82, appear to be correlated to lymph node metastasis in oesophageal cancer patients. As there are very few reports concerning MRP-1/CD9 or KAI1/CD82 expressions in intestinal tumours or oesophageal cancer, we compared our study with those on other cancers. MRP-1/CD9 was reported to reduce its expression in proportion to the state of lymph node metastasis in non-small cell lung cancer (Higashiyama et al, 1995), breast cancer (Miyake et al, 1995) and colon cancer (Cajot et al, 1997). Reduced KAI1/CD82 expression was reported to be correlated with lymph node metastasis and poor prognoses in non-small cell lung cancer, especially in adenocarcinoma but not in squamous cell carcinoma (Adachi et al 1996). We found compatible results indicating reduced expression of both MRP-1/CD9 and KAI1/CD82 correlated to lymph node metastasis in oesophageal squamous cell carcinoma. We could not, however, find an inverse correlation in distant metastatic cases. In these cases, almost all patients' distant metastases were cervical lymph node metastases. Finding that MRP-1/CD9 expression was inversely correlated to lymphatic invasion may suggest that this molecular loss might affect mainly the closest sites to the primary lesions and thus lead to the local lymph node metastases. Distant lymph node metastasis may require other changes.

Reduced expression also correlated to poor prognoses in the MRP-1/CD9 expression in oesophageal carcinoma, as they had in other carcinomas. As most of the oesophageal carcinoma showed reduced expressions of KAI1/CD82, the correlation between KAI1/CD82 expressions and prognoses is not presented here.

Although the mechanism of the inhibitory effect on metastasis of these two transmembrane proteins has not been examined extensively, the inhibitory effect on metastasis by MRP-1/CD9 is believed to be due to suppression of cell motility. MRP-1/CD9 was also reported to interact with heparin-binding epidermal growth factor (EGF)-like growth factor associated with $\alpha3\beta1$ integrin (Higashiyama et al, 1995; Nakamura et al, 1995). These adhesional effects may play an essential role in the initiation of a metastatic cascade. The antibody to MRP-1/CD9 was reported to aggregate platelets and activate their function (Hato et al, 1988; Higashihara et al, 1990). Platelets may release several growth factors which may facilitate tumour activation or growth.

We found a larger decrease in expression of KAI1/CD82 compared to MRP-1/CD9 in oesophageal cancer. Perhaps because of the specific nature of each antibody, the expression of KAI1/CD82 is thought to disappear at an earlier stage in carcinogenesis than MRP-1/CD9. Expression of both KAI1/CD82 and MRP-1/CD9 was preserved in many cases of dysplasia. The characteristic difference between the KAI1/CD82 and MRP-1/CD9 molecules may appear after dysplastic degradation in the oesophageal tumorigenesis.

KAI1/CD82 has a larger molecular weight, 29.610 kDa, than MRP-1/CD9, 24.28 kDa. Structurally, KAI1/CD82 has three *N*-glycosylation sites, while MRP-1/CD9 has one site (Boucheix C et al, 1991; Dong et al, 1995). It is thought that *N*-glycosylation sites play an important role in the suppression of metastasis (Hakomori et al, 1989), but it is not well known what role these differences play. Though there were some differences between them, their basic characteristics seemed to resemble each other. Their expression disappeared in oesophageal cancer and loss of that expression seemed to correlate with the ability to initiate metastasis. Preserving those molecules might prevent the patient from metastases and knowledge of the level of expression may become a useful predictor of prognoses for oesophageal cancer patients.

### ACKNOWLEDGEMENTS

I would like to thank Dr H Yamabe and the staff of the Department of Pathology of Kyoto University Hospital for their help and advice, to thank Miss Sandy Dzogan for proofreading this manuscript.

This work was supported in part by a grant-in-aid from the Japanese Ministry of Education, Science and Culture (grant C-2 07671386, B-07457271).

### REFERENCES

Adachi M, Taki T, Ieki Y, Huang CL, Higashiyama M and Miyake M (1996) Correlation of *KAI1/CD82* gene expression with good prognosis in patients with non-small cell lung cancer. *Cancer Res* **56**: 1751–1755

Boucheix C, Benoit P, Frachet P, Billard M, Worthington RE, Gagnon J and Uzan G (1991) Molecular cloning of the CD9 antigen. A new family of cell surface proteins. *J Biol Chem* **266**: 117–122

Cajot JF, Sordat I, Silvestre T and Sordat B (1997) Differential display cloning identifies motility-related protein (MRP-1/CD9) as highly expressed in primary compared to metastatic human colon carcinoma cells. *Cancer Res* **57**: 2593–2597

Dong JT, Lamb PW, Rinker-Schaeffer CW, Vukanovic J, Ichikawa T, Issac JT and Barrett JC (1995) *KAI1*, a metastasis suppressor gene for prostate cancer on human chromosome 11p11.2. *Science* **268**: 884–886

Dong JT, Suzuki H, Pin SS, Bova GS, Schalken JA, Issacs WB, Barrett JC and Issacs JT (1996) Down-regulation of the *KAI1* metastasis suppressor gene during the progression of human prostatic cancer infrequently involves gene mutation or allelic loss. *Cancer Res* **56**: 4387–4390

Hakomori S (1989) Aberrant glycolation in tumors and tumor-associated carbohydrate antigen. In *Advances in Cancer Research*, Vande-Woude GF and Klein G (eds), pp. 257–331. Academic Press: San Diego

Hato T, Ikeda K, Yasukawa M, Watanabe A and Kobayashi Y (1988) Exposure of platelet fibrinogen receptors by a monoclonal antibody to CD9 antigen. *Blood* **72**: 224–229

Hermanek P and Sobin LH (1987) TNM classification of malignant tumours, 4th edn. pp 40–42. Springer Verlag, Berlin

Higashihara M, Takahata K, Yatomi Y, Nakahara K and Kurokawa K (1990) Purification and partial characterization of CD9 antigen of human platelets. *FEBS* **264**: 270–274

Higashiyama M, Taki T, Ieki Y, Adachi M, Huang CL, Koh T, Kodama K, Doi O and Miyake M (1995) Reduced motility related protein-1 (*MRP-1/CD9*) gene expression as a factor of poor prognosis in non-small cell lung cancer. *Cancer Res* **55**: 6040–6044

Higashiyama S, Iwamoto R, Goishi K, Raab G, Taniguchi N, Klagsbrun M and Mekada E (1995) The membrane protein CD9/DRAP 27 potentiates the jutacrine growth factor activity of the membrane-anchored heparin-binding EGF-like growth factor. *J Cell Biol* **128**: 929–938

Ikeyama S, Koyama M, Yamaoko M, Sasada R and Miyake M (1993) Suppression of cell motility and metastasis by transfection with human motility-related protein (MRP-1/CD9) DNA. *J Exp Med* **177**: 1231–1237

Imamura M, Ohishi K, Mizutani N, Yanagibashi K, Naito M, Shimada Y, Hattori Y, Satomura K and Tobe T (1987) Retrosternal esophagogastrostomy with EEA stapler after subtotal resection of the esophagus: application and results. *Dig Surg* **4**: 101–105

Miyake M, Koyama M, Seno M and Ikeyama S (1991) Identification of the motility-related protein (MRP-1), recognized by monoclonal antibody M31-15, which inhibits cell motility. *J Exp Med* **174**: 1347–1354

© Cancer Research Campaign 1999

Miyake M, Nakano K, Ieki Y, Adachi M, Huang CL, Itoi S, Koh T and Taki T (1995) Motility-related protein 1 (MRP-1/CD9) expression: inverse correlation with metastases in breast cancer. *Cancer Res* **55**: 4127–4131

Nakamura K, Iwamoto R and Mekada E (1995) Membrane-anchored heparin-binding EGF-like growth factor (HB-EGF) and diphtheria toxin receptor-associated protein (DRAP 27)/CD9 form a complex with integrin α3β1 at cell–cell contact sites. *J Cell Biol* **129**: 1691–1705

Ueda T, Ichikawa T, Tamaru J, Mikata A, Akakura K, Akimoto S, Imai T, Yoshie O, Shiraishi T, Yatani R, Ito H and Shimazaki J (1996) Expression of the KAI1 protein in benign prostatic hyperplasia and prostate cancer. *Am J Pathol* **149**: 1435–1440

© Cancer Research Campaign 1999

## References

[PDF_00339] Adachi M., Taki T., Ieki Y., Huang C. L., Higashiyama M., Miyake M. (1996). Correlation of KAI1/CD82 gene expression with good prognosis in patients with non-small cell lung cancer.. Cancer Res.

[PDF_00342] Boucheix C., Benoit P., Frachet P., Billard M., Worthington R. E., Gagnon J., Uzan G. (1991). Molecular cloning of the CD9 antigen. A new family of cell surface proteins.. J Biol Chem.

[PDF_00345] Cajot J. F., Sordat I., Silvestre T., Sordat B. (1997). Differential display cloning identifies motility-related protein (MRP1/CD9) as highly expressed in primary compared to metastatic human colon carcinoma cells.. Cancer Res.

[PDF_00349] Dong J. T., Lamb P. W., Rinker-Schaeffer C. W., Vukanovic J., Ichikawa T., Isaacs J. T., Barrett J. C. (1995). KAI1, a metastasis suppressor gene for prostate cancer on human chromosome 11p11.2.. Science.

[PDF_00352] Dong J. T., Suzuki H., Pin S. S., Bova G. S., Schalken J. A., Isaacs W. B., Barrett J. C., Isaacs J. T. (1996). Down-regulation of the KAI1 metastasis suppressor gene during the progression of human prostatic cancer infrequently involves gene mutation or allelic loss.. Cancer Res.

[PDF_00359] Hato T., Ikeda K., Yasukawa M., Watanabe A., Kobayashi Y. (1988). Exposure of platelet fibrinogen receptors by a monoclonal antibody to CD9 antigen.. Blood.

[PDF_00367] Higashiyama M., Taki T., Ieki Y., Adachi M., Huang C. L., Koh T., Kodama K., Doi O., Miyake M. (1995). Reduced motility related protein-1 (MRP-1/CD9) gene expression as a factor of poor prognosis in non-small cell lung cancer.. Cancer Res.

[PDF_00371] Higashiyama S., Iwamoto R., Goishi K., Raab G., Taniguchi N., Klagsbrun M., Mekada E. (1995). The membrane protein CD9/DRAP 27 potentiates the juxtacrine growth factor activity of the membrane-anchored heparin-binding EGF-like growth factor.. J Cell Biol.

[PDF_00375] Ikeyama S., Koyama M., Yamaoko M., Sasada R., Miyake M. (1993). Suppression of cell motility and metastasis by transfection with human motility-related protein (MRP-1/CD9) DNA.. J Exp Med.

[PDF_00382] Miyake M., Koyama M., Seno M., Ikeyama S. (1991). Identification of the motility-related protein (MRP-1), recognized by monoclonal antibody M31-15, which inhibits cell motility.. J Exp Med.

[PDF_00389] Miyake M., Nakano K., Ieki Y., Adachi M., Huang C. L., Itoi S., Koh T., Taki T. (1995). Motility related protein 1 (MRP-1/CD9) expression: inverse correlation with metastases in breast cancer.. Cancer Res.

[PDF_00393] Nakamura K., Iwamoto R., Mekada E. (1995). Membrane-anchored heparin-binding EGF-like growth factor (HB-EGF) and diphtheria toxin receptor-associated protein (DRAP27)/CD9 form a complex with integrin alpha 3 beta 1 at cell-cell contact sites.. J Cell Biol.

[PDF_00397] Ueda T., Ichikawa T., Tamaru J., Mikata A., Akakura K., Akimoto S., Imai T., Yoshie O., Shiraishi T., Yatani R. (1996). Expression of the KAI1 protein in benign prostatic hyperplasia and prostate cancer.. Am J Pathol.

